# Dementia in the older population is associated with neocortex content of serum amyloid P component

**DOI:** 10.1093/braincomms/fcab225

**Published:** 2021-10-09

**Authors:** Stephan Ellmerich, Graham W Taylor, Connor D Richardson, Thais Minett, Amand Floriaan Schmidt, Carol Brayne, Fiona E Matthews, Paul G Ince, Stephen B Wharton, Mark B Pepys, Cognitive Function and Ageing Study, Carol Brayne, Fiona E Matthews, Louise Robinson, Adelina Comas-Herrera, Bob Woods, Blossom Stephan, Bronwyn Parry, Carol Jagger, Linda Clare, Tom Dening, Linda Barnes, Gill Forster, Ian McKeith, Raphael Wittenberg, Stephen B Wharton, Sarah Pendlebury, Simon Harrison, Antony Arthur, Roy Weller, Stuart Pickering-Brown, Paul G Ince

**Affiliations:** Wolfson Drug Discovery Unit, UCL Royal Free Campus, London NW3 2PF, UK; Wolfson Drug Discovery Unit, UCL Royal Free Campus, London NW3 2PF, UK; Population Health Sciences Institute; Newcastle University, Newcastle upon Tyne NE4 5PL, UK; Department of Radiology, Cambridge Biomedical Research Centre, University of Cambridge, Cambridge CB2 0QQ, UK; Institute of Cardiovascular Science, University College London, London WC1E 6DH, UK; Cambridge Public Health, Cambridge CB2 1PZ, UK; Population Health Sciences Institute; Newcastle University, Newcastle upon Tyne NE4 5PL, UK; Sheffield Institute for Translational Neuroscience, University of Sheffield, Sheffield S10 2HQ, UK; Sheffield Institute for Translational Neuroscience, University of Sheffield, Sheffield S10 2HQ, UK; Wolfson Drug Discovery Unit, UCL Royal Free Campus, London NW3 2PF, UK

**Keywords:** serum amyloid P component, dementia, neocortex

## Abstract

Despite many reported associations, the direct cause of neurodegeneration responsible for cognitive loss in Alzheimer’s disease and some other common dementias is not known. The normal human plasma protein, serum amyloid P component, a constituent of all human fibrillar amyloid deposits and present on most neurofibrillary tangles, is cytotoxic for cerebral neurones *in vitro* and in experimental animals *in vivo*. The neocortical content of serum amyloid P component was immunoassayed in 157 subjects aged 65 or more with known dementia status at death, in the large scale, population-representative, brain donor cohort of the Cognitive Function and Ageing Study, which avoids the biases inherent in studies of predefined clinico-pathological groups. The serum amyloid P component values were significantly higher in individuals with dementia, independent of serum albumin content measured as a control for plasma in the cortex samples. The odds ratio for dementia at death in the high serum amyloid P component tertile was 5.24 (95% confidence interval 1.79–15.29) and was independent of Braak tangle stages and Thal amyloid-β phases of neuropathological severity. The strong and specific association of higher brain content of serum amyloid P component with dementia, independent of neuropathology, is consistent with a pathogenetic role in dementia.

## Teaser

The normal blood protein, serum amyloid P component (SAP), is toxic for cerebral neurones but normally is largely excluded from the brain. Ellmerich et al. report that higher neocortical content of SAP is strongly associated with dementia at death, consistent with a pathogenetic role of SAP in neurodegeneration.

## Graphical Abstract

**Graphical Abstract:**
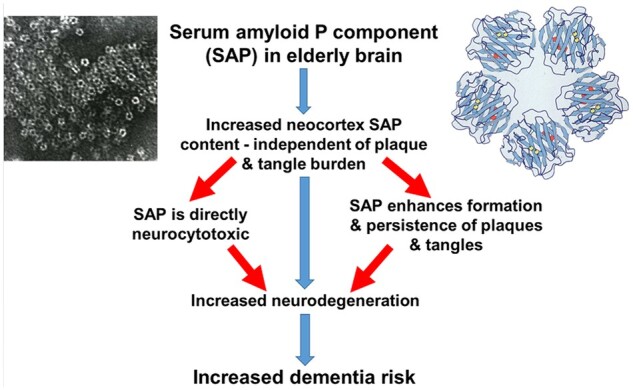

## Introduction

The cognitive loss that characterizes Alzheimer’s disease and other common dementias is caused by neurodegeneration, often associated with cerebral small vessel disease, but the processes directly responsible for dysfunction and death of cerebral neurones are poorly understood. Serum amyloid P component (SAP), a normal plasma protein which is a trace constituent of CSF,^1^^,^^2^ has been reported to be neurotoxic for cerebral neurones *in vitro*^3–5^ and *in vivo*.^6^ In these experimental studies, SAP binds to the neuronal surface, is internalized, traffics to the nucleus^7^ where it binds avidly to chromatin^8^ and triggers apoptotic cell death.^4^^,^^9^ These processes may also occur *in vivo* in humans, as SAP is detectable by immunohistochemistry within some cerebral neurones even in normal adult brain (MB Pepys, unpublished observations). Furthermore, SAP is a universal constituent of human amyloid deposits, including the Aβ amyloid deposits of Alzheimer’s disease and cerebral amyloid angiopathy (CAA), and SAP is also present on most neurofibrillary tangles (NFTs).^10–12^ This is due to the avid but reversible, specific, calcium-dependent binding of SAP to all amyloid fibril types and to the hyperphosphorylated tau of NFT.^2^ Bound SAP supports both formation^13^^,^^14^ and persistence^15^ of Aβ amyloid fibrils and may similarly stabilize tangles.^2^ SAP may thus contribute to pathogenesis of dementia by two distinct pathways, direct cerebral neurotoxicity and enhancement of plaque and tangle pathology, and is accordingly a valid therapeutic target.^2^^,^^16^

Here, we report the first robustly scaled investigation of the relationship between dementia and brain SAP content. SAP was assayed in samples of neocortex from the brains of 157 participants in the Cognitive Function and Ageing Study (CFAS), whose dementia status at death was known and whose neuropathology was comprehensively documented.

## Materials and methods

### The Cognitive Function and Ageing Study

CFAS is a longitudinal study of cognitive impairment and frailty in the UK population aged 65 years and older (www.cfas.ac.uk),^17^ which includes a population-representative brain donation cohort. Dementia status of CFAS participants was established using multiple information sources, including Automated Geriatric Examination for Computer Assisted Taxonomy (AGECAT), notification of dementia in death certificates, a Retrospective Informant Interview (RINI; www.cfas.ac.uk) with relatives and carers after death, and the probability of having dementia before death from a Bayesian analysis of all individuals modelling the prevalence and incidence of dementia in CFAS. We could not assign dementia status in 30 individuals in whom the study diagnosis was ‘no dementia’. These respondents were not included in the analysis because their last interview was more than 6 months before death, no RINI was available, and dementia was not mentioned on the death certificate. Dementia status was known for a total of 507 participants in CFAS, of whom 257 were from the Cambridge, Oxford and Newcastle Centres that have the neuropathological material available. SAP was measured in the 157 subjects, comprising 60 men and 97 women, whose stored brains included frozen frontal and/or temporal neocortex. As a control for the presence of plasma as the source of the SAP detected, serum albumin was also measured in 69 frontal cortex samples from subjects with known dementia status. Dementia status was known for the donors of 154 temporal and 128 frontal neocortex samples. No hippocampus was available.

### Tissue processing and serum protein immunoassays

Samples of temporal (Brodmann Area 21, *n* = 154) and/or frontal neocortex (Brodmann Area 8/9, *n* = 128) were expertly cryo-dissected to remove subcortical white matter and arachnoid mater and a known mass of each was suspended in 2 ml chilled 10 mM Tris buffered 140 mM NaCl at pH 8.0 containing 10 mM ethylene diamine tetraacetic acid, 320 mM sucrose and 0.5% v/v triton X-100, to which 1% v/v Sigma P8340 protease inhibitors (dimethyl sulphoxide solution of 104 mM 4-(2-aminoethyl)benzenesulfonyl fluoride hydrochloride; 80 μM aprotinin; 4 mM bestatin; 1.4 mM E-64; 2 mM leupeptin; 1.5 mM pepstatin A) was added immediately before use. Inclusion of ethylene diamine tetraacetic acid is essential for elution of SAP from its strictly calcium dependent binding to amyloid fibrils and NFT. The tissue was promptly homogenized (TissueLyser, Qiagen) at 50 Hz for 4 min, then centrifuged at 10 600 *g* for 15 min and the supernatant removed for storage at −30°C until assayed. The SAP content was determined by a robustly reproducible immunoradiometric assay,^18^ calibrated with isolated pure human SAP.^19^ There was complete recovery of SAP added to control cortex samples before homogenization, confirming that the tissue processing did not affect SAP quantification. SAP content is expressed as ng/mg of neocortex tissue. Serum albumin was assayed in homogenates by enzyme-linked immunosorbent assay (Merck) calibrated precisely with standards of 99% pure human serum albumin (Merck) quantified by A_280_ using the specific 1%, 1 cm extinction coefficient, 6.54, and expressed as µg/mg neocortex tissue. All tissue processing and immunoassays were conducted on coded samples, blind to all subject information.

### Statistical analysis

Only one cortical region was available in 32 subjects, 3 frontal and 29 temporal, while both regions were available in 125 subjects. For statistical analysis as the overall neocortical SAP content, and designated as such, the higher of the two values was adopted when both were available and the single available value was used in the other subjects. Based on associations in previous CFAS analysis,^20^ modelling of effects on dementia risk was appropriate for seven neuropathological variables. Braak stage, Thal phase and CAA were included in the final model, but the other variables (brain weight, age at death, histological/imaging vascular disease, hippocampal atrophy and Lewy bodies) were excluded due to diminished sample size and statistical power.

Differences in mean SAP by dementia status were assessed using the Kruskal–Wallis test for continuous SAP variables and the chi-square test for categorical analysis. Pairwise comparison of means was used to assess SAP by dementia status for individual Braak stages and Thal phases, with *P*-values adjusted for multiple comparisons by Tukey’s method.

Logistic regression models were used to analyse the relationship between SAP content and dementia risk, adjusted for the known neuropathology variables.

Neocortex content of SAP and albumin was analysed by Spearman’s correlation coefficient with *R*^2^ representing the explained variance.

### Data availability

The data from this study are archived in the CFAS Neuropathology Data Archive and are available using the data application form.

## Results

### Dementia is associated with neocortex SAP content independently of plaque and tangle burden

**Figure 1:**
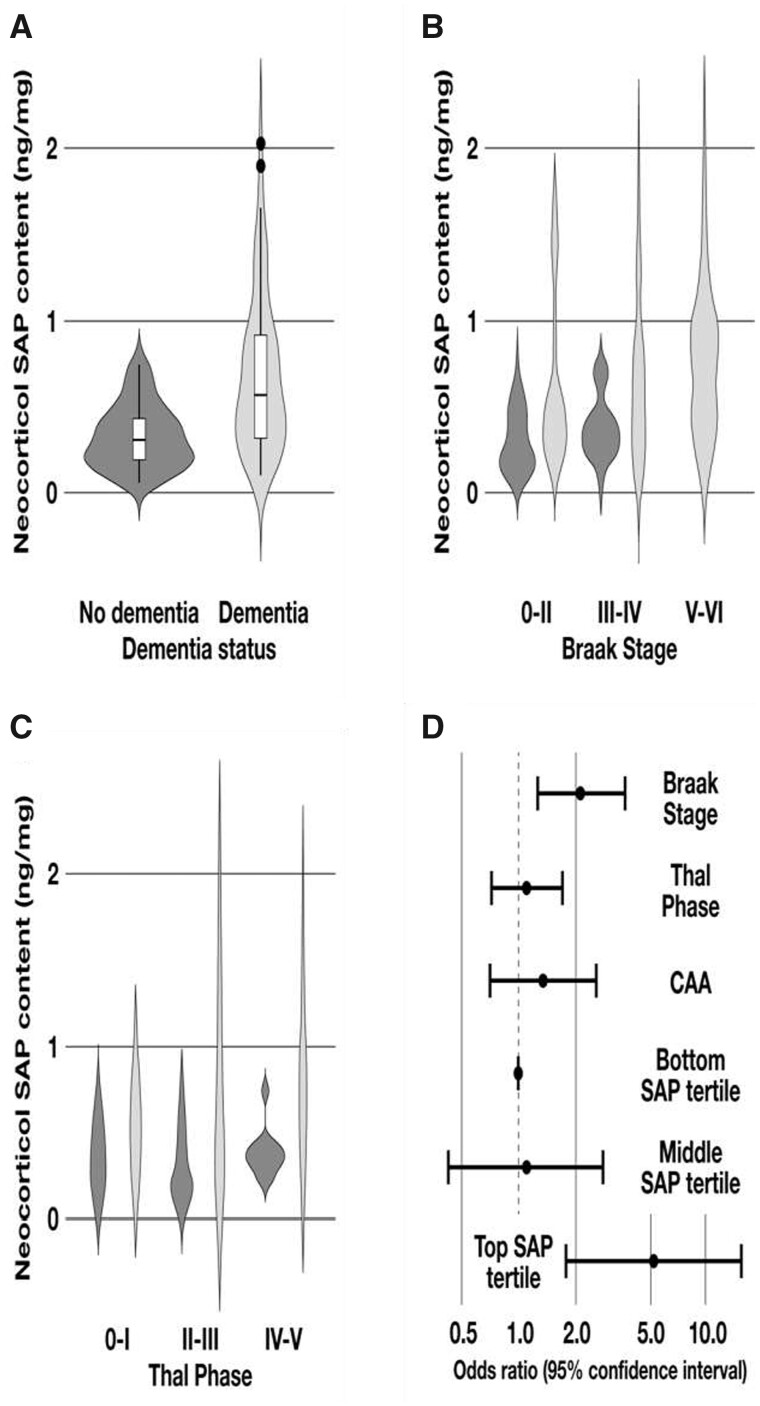
Descriptive statistics and logistic regression modelling of the relationships between neocortical SAP content, dementia and neuropathological staging. (**A**) Distribution of SAP values in subjects with and without dementia, illustrated by violin plots showing the higher median in those with dementia, the interquartile range, the lower/upper adjacent values and outliers. (**B**) Distribution of SAP values in subjects with and without dementia with increasing severity of Braak stages. (**C**) Distribution of SAP values in subjects with and without dementia with increasing severity of Thal phases. (**D**) Risk of dementia according to SAP values, adjusted for classical dementia neuropathology.

The mean and median neocortical SAP content values were significantly higher in subjects who had dementia at death than in those who did not, despite substantial overlap in the respective distributions (Table 1, Fig. 1A). Since SAP is always present in intracerebral and cerebrovascular Aβ amyloid deposits and on most NFT, the neocortical SAP content might simply reflect the total burden of amyloid and tangles. Indeed, there is a correlation between the SAP values and both the Braak NFT stages and the Thal Aβ phases. However, within each of these neuropathological categories, the median SAP content was higher in those with dementia (Fig. 1B and C). Table 1 shows the risk of dementia at death according to cortical SAP content, modelled by logistic regression analysis both with and without adjustment for the Braak and Thal classifications and the CAA scores (Fig. 1D). SAP content was independently related to dementia at death, with odds ratio (OR) of 5.24 [95% confidence interval (CI) 1.79–15.29] for the high SAP tertile. Dementia was thus more closely associated with brain SAP content than with classical neuropathology.

**Table 1:** Mean neocortical SAP content (ng/mg tissue) by region and dementia status

		No dementia		Dementia				Unadjusted^a^		Adjusted^a^	
	*n*	*n*	Mean	*n*	Mean	*t*	*P*	ORb	95% CIc	ORb	95% CIc
Temporal SAP content (*n* = 154)	154	65	0.30	89	0.55	4.76	<0.01	1.38	(1.18, 1.61)	1.34	(1.12, 1.61)
Frontal SAP content (*n* = 128)	128	51	0.25	77	0.54	4.98	<0.01	1.50	(1.24, 1.81)	1.38	(1.13, 1.70)
Neocortical SAP content (*n* = 157)	157	66	0.33	91	0.66	5.85	<0.01	1.45	(1.25, 1.70)	1.38	(1.15, 1.65)
Tertiles of neocortical SAP content											
Low	52	32	0.17	20	0.21	4.88	<0.01	Reference^d^		Reference^d^	
Middle	53	25	0.41	28	0.42			1.79	(0.82, 3.90)	1.10	(0.43, 2.83)
High	52	9	0.65	43	1.02			7.64	(3.08, 18.99)	5.24	(1.79, 15.29)
Braak stage	135									2.15	(1.26, 3.67)
0–II	50	32	0.30	18	0.58	2.64	<0.01 (0.03)				
III–IV	56	21	0.38	35	0.65	2.67	<0.01 (0.03)				
V–VI	29	2	0.23	27	0.78	2.09	0.04 (0.11)				
Thal phase	138									1.11	(0.72, 1.71)
0–I	30	18	0.35	12	0.52	1.28	0.20 (0.61)				
II–III	55	27	0.29	28	0.61	3.33	<0.01 (<0.01)				
IV–V	53	11	0.38	42	0.77	3.32	<0.01 (<0.01)				

^a^ With respect to neuropathological categories.

^b^ OR, odds ratio.

^c^ CI, confidence interval.; *P*-values in parentheses show Tukey multiple comparisons correction.

^d^ The low tertile of the neocortical SAP content distribution was used as the reference category, corresponding to odds ratio of 1.

### The SAP–dementia relationship is independent of other neuropathology variables

The prevalence of different neuropathology features among the study population is shown in Table 2 according to their dementia status. Sensitivity analyses including each neuropathology variable univariately, and using a multivariable model, showed that they did not influence the relationship of SAP to dementia. In addition to the model of SAP adjusted for Braak stages, Thal phases and CAA, Table 3 shows the OR and 95% CI for SAP content and dementia risk adjusted for each of the neuropathology variables.

**Table 2:** Neocortex SAP content, neuropathology and dementia status

Neuropathology variables	Severity	**No dementia** ^a^	**Dementia** ^a^	Status unknown^a^
		*n* = 66 (36.67)	*n* = 91 (50.56)	*n* = 23 (12.78)
Temporal SAP tertiles	Low	30 (46.15)	24 (26.97)	5 (23.81)
	Middle	24 (36.92)	26 (29.21)	8 (38.10)
	High	11 (16.92)	39 (43.82)	8 (38.10)
Frontal SAP tertiles	Low	28 (24.90)	15 (19.48)	7 (35.00)
	Middle	15 (29.41)	26 (33.77)	8 (40.00)
	High	8 (15.69)	36 (46.75)	5 (25.00)
Neocortical SAP	Low	32 (48.48)	20 (21.98)	8 (34.78)
	Moderate	25 (37.88)	28 (30.77)	7 (30.43)
	High	9 (13.64)	43 (47.25)	8 (34.78)
Thal phase	0–I	18 (32.14)	12 (14.63)	3 (33.33)
	II–III	27 (48.21)	28 (34.15)	4 (44.44)
	IV–V	11 (19.64)	42 (51.22)	2 (22.22)
Braak stage	0–II	32 (58.18)	18 (22.50)	4 (57.14)
	III–IV	21 (38.18)	27 (43.86)	3 (24.86)
	V–VI	2 (3.64)	27 (33.75)	0
Lewy bodies	Not present	63 (35.45)	74 (81.32)	10 (90.91)
	Present	3 (4.55)	17 (18.68)	1 (9.09)
Brain weight (kg)	Median	1.19	1.12	1.17
Age at death (y)	<80	22 (33.33)	8 (8.79)	3 (25.00)
	81–89	31 (46.97)	47 (51.65)	7 (58.33)
	>89	13 (19.70)	36 (39.56)	2 (16.67)
CAA	None/mild	30 (53.57)	24 (29.27)	5 (55.56)
	Moderate	19 (33.93)	33 (40.24)	2 (22.22)
	Severe	7 (15.50)	25 (30.49)	2 (22.22)
Histological/imaging vascular disease	None	8 (14.81)	9 (12.00)	1 (16.67)
	Infarct or haemorrhage	2 (3.70)	2 (2.67)	0
	Lacunes/SVD^b^/DWML^c^	35 (64.81)	38 (50.67)	2 (33.33)
	Both	9 (16.67)	26 (34.67)	3 (50.00)
Hippocampal atrophy	None	5 (13.89)	5 (6.94)	1 (16.67)
	Mild	20 (55.56)	23 (32.94)	4 (66.67)
	Moderate	11 (30.56)	34 (47.22)	1 (16.67)
	Severe	0	10 (13.89)	0

a All results are shown as number of individuals (percentage of total in all groups).

b Small vessel disease.

c Deep white matter lesions.

**Table 3:** Logistic regression model of neocortical SAP content and dementia risk

Neuropathology^a^	OR^b^	**95% CI** ^c^
Neocortical SAP	1.39	(1.02–1.88)
Thal phase	1.25	(0.54–2.91)
Braak stage	1.88	(0.76–4.63)
Lewy bodies	15.98	(0.80–318.66)
Brain weight (kg)	1.00	(0.99–1.00)
Age at death (years)	1.17	(1.03–1.33)
Cerebral amyloid angiopathy	0.73	(0.17–3.20)
Histological/imaging vascular disease	2.02	(0.70–5.78)
Hippocampal atrophy	1.42	(0.50–4.03)

a Neuropathology variables used to adjust OR for SAP and dementia risk.

b Odds ratio.

c 95% confidence intervals.

### Neocortex content of SAP and of serum albumin are unrelated

The concentration of SAP in plasma is about 1000-fold higher than in the CSF^1^^,^^21^ and the amount of SAP in the homogenates could therefore be influenced by the presence of plasma in the neocortex samples. However, the square of Spearman’s coefficient for the amounts of SAP and albumin in the frontal cortex samples, *R*^2^, was 0.05, showing that the albumin content explains less than 6% of the variance in SAP content (Supplementary Fig. 1). The SAP detected in the samples was thus unlikely to be derived from the incidental presence of plasma and was the content of the cortex itself. There was no significant difference between the neocortical albumin content of individuals with (*n* = 46) and without dementia at death (*n* = 23); *P *=* *0.91 for pairwise comparison of means.

## Discussion

We show here, using the CFAS neuropathology cohort, that dementia is related to brain SAP content. Unsurprisingly, since SAP is bound to all Aβ amyloid deposits and to most NFTs, brain SAP content shows some correlation with these neuropathological markers of Alzheimer’s. However, we demonstrate that *within* given stages of Alzheimer’s neuropathological change, neocortical SAP content is higher in those with dementia. Furthermore, the SAP–dementia relationship is independent of other neuropathological variables in a logistic regression model, indicating a relationship of SAP to dementia that is independent of classical neuropathological lesions and is consistent with a pathogenic role.

Studies in CFAS, and other population-based neuropathology studies, have demonstrated that classical neurodegenerative and vascular neuropathological lesions do not account for all of dementia in the elderly population, so that other factors may contribute to or modulate dementia.^17^ The population-representative brain donation cohort within this longitudinal study of cognitive impairment and frailty in the population aged 65 years and older (www.cfas.ac.uk),^17^ crucially enabled the present separate assessment of the relationship of SAP content to dementia and to neuropathology thus avoiding the biases inherent in using clinico-pathological groups which have been pre-defined by combining neuropathological measures with clinical dementia status. The only previous study of brain SAP content and dementia comprised pre-selected groups: 8 subjects with Alzheimer’s disease, 6 with Alzheimer’s disease neuropathology but not dementia, 5 with mild cognitive impairment and 9 controls.^22^ It suggested an association between cortical SAP content and dementia that we here robustly confirm and extend.

Major strengths of the present study are its geographically and population-based data capture linked to a representative donor programme, its longitudinal nature, robust and standardized approaches to establishment of clinical status before death, and careful post-mortem donation process allowing storage for new data creation. Although we have studied almost six times more subjects than the only previous investigation of cortical SAP content, our sample size is still limited. In addition, the hippocampus was not available although the key frontal and temporal neocortex were included. Furthermore, the only obvious confounder, the presence of plasma within the neocortex samples, was eliminated by assay of serum albumin in a substantial subset.

Circulating SAP is synthesized and catabolized only by the liver and is not present in the normal adult human brain transcriptome.^23^ Claims for local cerebral expression of SAP, based on mRNA detection, have not been substantiated by direct measurements^24^; furthermore, depletion of SAP from the blood in patients with Alzheimer’s disease completely removed SAP from the CSF.^2^ SAP thus only enters the brain from the blood and the SAP concentration in the CSF is about one-thousandth of the plasma concentration, a much lower ratio than for other comparable plasma proteins. In addition to exclusion by the blood–brain barrier, SAP may even be actively transported across the barrier and out of the brain.^25^ However, the age-associated decrease of blood–brain barrier efficiency^26^ may attenuate protection of the brain against SAP neurotoxicity. CFAS has reported that the association of dementia with the neocortical neuritic plaque burden decreases with age whilst the association with cortical atrophy is unaffected.^27^ This is consistent with the key role of neuronal and/or synaptic loss in dementia and with a possible contribution of direct SAP neurotoxicity, rather than other effects of plaques and tangles. Furthermore, cerebral haemorrhage, traumatic brain injury and severe or repeated non-penetrating head injury, as in boxers and football players, are associated with higher dementia risk. All these conditions compromise the blood–brain barrier and/or enable direct overexposure of cerebral neurones to circulating SAP.

Our present measurements of albumin content in the neocortex samples show that the quantities of SAP that are present, and that correlate with dementia status, are not just the result of non-specific leakage through the blood barrier. Despite the ∼100 000-fold greater abundance of albumin than SAP in the CSF,^1^^,^^2^ the amount of SAP relative to albumin was much greater in all neocortex samples, consistent with specific retention and persistence of SAP in the tissue.

SAP binds avidly and stably to both Aβ amyloid fibrils and NFT but the association of human dementia with neocortex SAP content was independent of the abundance of cerebral plaque and cerebrovascular amyloid as well as NFTs. The association is therefore consistent with a direct pathogenic role of SAP in neurodegeneration, which can only be definitively established *in vivo* by a specific therapeutic intervention. The safe and well tolerated drug, miridesap, hexanoyl bis(D-proline) previously known as CPHPC, depletes circulating SAP by over 90% for as long as the drug is administered^16^^,^^28^ and thereby removes all SAP from the CSF^2^ and from intracerebral and cerebrovascular amyloid deposits.^29^ The ongoing DESPIAD (Depletion of serum amyloid P component in Alzheimer’s disease), double-blind, placebo controlled, 1 year, phase 2b clinical trial of SAP depletion by miridesap in patients with Alzheimer’s disease, is monitoring cortical atrophy, cerebral amyloid burden, cognition and multiple other blood and CSF biomarkers including neurofilament light chain, aiming to detect any significant effects on disease progression (https://www.clinicaltrialsregister.eu/ctr-search/trial/2016-003284-19/GB).

## Supplementary material

Supplementary material is available at *Brain Communications* online.

## Supplementary Material

fcab225_Supplementary_Data

## Appendix

**CFAS Neuropathology Management Committee:** Carol Brayne; Fiona E. Matthews; Louise Robinson; Adelina Comas-Herrera; Bob Woods; Blossom Stephan; Bronwyn Parry; Carol Jagger; Linda Clare; Tom Dening; Linda Barnes; Gill Forster; Ian McKeith; Raphael Wittenberg; Stephen B. Wharton; Sarah Pendlebury; Simon Harrison; Antony Arthur; Roy Weller, Stuart Pickering-Brown; Paul G. Ince.

## Abbreviations

AGECAT: = Automated Geriatric Examination for Computer Assisted Taxonomy
CAA: = cerebral amyloid angiopathy
CFAS: = Cognitive Function and Ageing Study
CI: = confidence interval
NFT: = neurofibrillary tangle
OF: = odds ratio
RINI: = Retrospective Informant Interview
SAP: = serum amyloid P component
