# Supplementary figures and images for: Dementia in the older population is associated with neocortex content of serum amyloid P component
Source: Brain Commun. 2021 Oct 9;3(4):fcab225. doi: 10.1093/braincomms/fcab225 (PMC8523881; doi:10.1093/braincomms/fcab225)

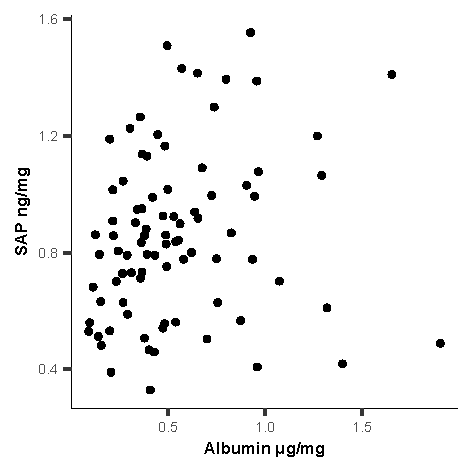

Supplement: fcab225_Supplementary_Data [file fcab225_supplementary_data.zip › fcab225_Supplementary_Data.tif]
